# Understanding American tegumentary leishmaniasis in urban Montes Claros, Brazil: insights from clinical, immunological and therapeutic investigations

**DOI:** 10.1017/S0031182024001057

**Published:** 2024-09

**Authors:** Dayse M. S. Lopes, Jackeline M. S. Lima, Karine S. M. Ribeiro, Clarissa F. Gomes, Rebeca M. Rocha, Thainara S. Gonçalves, Thallyta M. Vieira, Sílvio F. de Carvalho, M. G. Finn, Ana Paula Venuto, Alexandre F. Marques

**Affiliations:** 1 Universidade Estadual de Montes Claros – Unimontes, Montes Claros, MG, Brazil; 2 School of Chemistry and Biochemistry, Georgia Institute of Technology, Atlanta, GA, USA; 3 Center for Molecular and Cellular Biosciences, School of Biological, Environmental, and Earth Sciences, University of Southern Mississippi, Hattiesburg, MS, USA

**Keywords:** alpha-gal, American tegumentary leishmaniasis, *Leishmania braziliensis*, *Leishmania infantum*

## Abstract

The challenge of American tegumentary leishmaniasis (ATL) continues in Brazil, presenting a persistent public health issue despite initiatives aimed at public outreach, vector control and health education. To gain a deeper understanding of this disease, a study was conducted in an endemic region located in the northern region of the state of Minas Gerais, Brazil. The study monitored 30 resident patients diagnosed with ATL, using serum samples from 6 healthy individuals as controls. The localized cutaneous form of the disease was found to be predominant, with lesions appearing on various parts of the body and the majority of the affected individuals being male. The study found significantly higher levels of IgG anti-*α*-Gal antibodies in ATL-infected patients compared to healthy individuals. Treatment of 19 patients with meglumine antimoniate resulted in limited improvement in symptoms for most. Nonetheless, the study found that 12 patients who completed treatment with epithelialization of the lesions showed a significant decrease in IgG anti-*α*-Gal antibodies, indicating potential applications of this antibody in the diagnosis and monitoring of the disease. The study also identified *Leishmania* species in 7 analysed patients, revealing 6 cases infected by *Leishmania braziliensis* and 1 by *L. infantum*, with a significant difference in the anti-*α*-Gal responses. The findings of the study emphasize the urgent need for the development of human vaccines and innovative treatment strategies adapted to the diversity of *Leishmania* species causing cutaneous leishmaniasis and individual patient responses to improve the clinical management of ATL in Brazil and similar endemic regions.

## Graphical abstract

**Figure:**
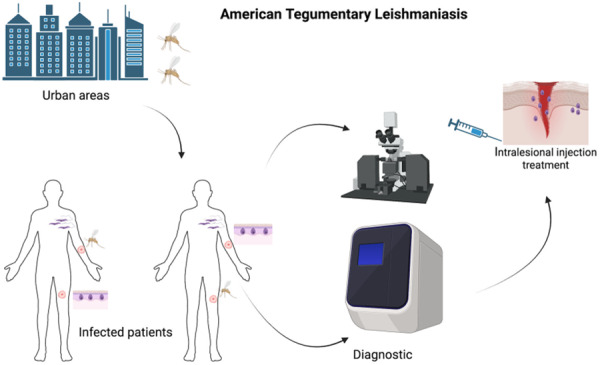

## Introduction

Leishmaniasis is an infectious disease that affects humans and other mammals and is caused by infection with the intracellular protozoa of the genus *Leishmania* (Ross, 1903). These parasites are transmitted during the blood meal of female phlebotomine insects of the genera *Lutzomyia* for New World Latin America and *Phlebotomus* for the Old-World Mediterranean Basin, Asia, and parts of Africa (Yamey and Torreele, 2002; Chappuis *et al*., 2007). Leishmaniasis has numerous clinical manifestations and different therapeutic responses (Machado *et al*., 2019). Individual clinical presentations are affected by factors such as age, nutritional status, *Leishmania* species and the individual's immune capacity (Kevric *et al*., 2015). Two primary clinical forms of the disease have been identified: visceral leishmaniasis (VL), also known as kalazar (the most severe form), and cutaneous or tegumentary leishmaniasis (TL), which is the most common form (Soong *et al*., 2012; Pasha *et al*., 2022).

According to the World Health Organization (WHO) (WHO, 2024), it is estimated that between 0.7 and 1 million new cases of TL occur annually worldwide, with the Americas being the continent responsible for more than 95% of the cases (Sheikh *et al*., 2024). It is known in the Americas as American tegumentary leishmaniasis (ATL), a non-contagious parasitic dermatological condition (Akhoundi *et al*., 2016) is mainly associated with poverty in developing countries (Burza *et al*., 2018), ATL is regarded as a neglected tropical disease. Although rarely fatal, ATL can severely harm the patient's daily life by causing destructive, disfiguring and disabling ulcerative lesions on the skin and mucous membranes. The severity of manifestation can vary from localized cutaneous leishmaniasis, showing single or multiple lesions in the same location that may heal spontaneously, to ulcerative forms that are much more severe. Even in its mildest form, disease healing can take several months and leave disfiguring scars (Pearson and Sousa, 1996). Approximately 40 million people worldwide suffer from stigmatizing effects caused by inactive scars of the disease (Bailey *et al*., 2017; 2019; Bennis *et al*., 2017).

The immunological status plays a significant role in the presentation and development of leishmaniasis. The disseminated form is characterized by numerous papular and acneiform lesions (acne-like) involving various body parts owing to haematogenous or lymphatic distribution (Machado *et al*., 2019). Diffuse cutaneous leishmaniasis occurs in patients without known immunodeficiency causes, lacks a specific cellular response to *Leishmania* antigens, and is characterized by the formation of diffuse non-ulcerated lesions throughout the skin. In mucosal/mucocutaneous leishmaniasis, the immune response is exacerbated and ineffective, affecting nasopharyngeal regions by destroying infected tissues (Silveira *et al*., 2004; Volpedo *et al*., 2021).

For leishmaniasis treatment, meglumine antimoniate, a pentavalent antimony derivative available in the Americas, has been advocated as the first choice drug in Brazil. It can be administered *via* intramuscular (IM), intravenous (IV) or intralesional (IL) injection. This medication has severe and well-known adverse effects, including cardiac, hepatic, pancreatic, renal and musculoskeletal system toxicities, making its use contraindicated in patients with relevant comorbidities (Sampaio *et al*., 2019). Another option in Brazil is miltefosine, administered orally with few side effects, most commonly discrete changes in kidney and liver function, nausea and vomiting (Machado *et al*., 2010).

Once considered a rural disease, leishmaniasis is now expanding to urban areas and involves several variables. In Brazil, these include a large population of canine reservoirs, the presence and adaptation of vectors, climate changes and human migration (Harhay *et al*., 2011; Rocklov and Dubrow, 2020; Mojahed *et al*., 2022). However, this new urban and peri-urban leishmaniasis migration has been observed not only in Brazil (Jeronimo *et al*., 2004) but also in countries such as Italy (Tarallo *et al*., 2010), Iran (Oshaghi *et al*., 2010), Mexico (Sanchez-Garcia *et al*., 2010) and Morocco (Boussaa *et al*., 2005). This epidemiological change in disease ecology and the migration of infected people to cities creates new opportunities for outbreaks in traditionally non-endemic regions of Europe and North America and spread to new sites in already endemic countries. Climate change also has the potential to progressively create ideal settings in Europe and North America for sandfly vectors (Boussaa *et al*., 2005; Ready, 2008; Gonzalez *et al*., 2010).

The infective promastigote form of *Leishmania* has a glycocalyx on its surface, composed mostly of molecules fixed by a glycosylphosphatidylinositol (GPI) anchor. Among these surface glycoconjugates are lipophosphoglycans, proteophosphoglycans and glycoinositolphospholipids (McConville *et al*., 1990; Assis *et al*., 2012). These have been shown to contribute to parasite virulence, infectivity, survival and pathogenicity (Gupta *et al*., 2022) by modulating essential functions related to the parasite/host interaction, including the invasion of cells that promote the host's innate immune response (Descoteaux and Turco, 1999; Assis *et al*., 2012; Carneiro and Peters, 2021). At the structural level, the *α*-Gal trisaccharide epitope (Gala1-3Galb1-4GlcNAc-) has been identified as a key carbohydrate present at varying levels on the surfaces of *Leishmania major*, *Leishmania infantum* and *Leishmania amazonensis* (Al-Salem *et al*., 2014; Moura *et al*., 2017). In this study, we investigated the production of anti-*α*-Gal antibodies by 30 patients diagnosed with ATL in the urban setting of Montes Claros-MG, Brazil, correlating this molecular marker with sociodemographic factors, clinical characteristics and treatment. Our findings provide valuable insights into ATL's demographic and clinical aspects and the potential significance of anti-*α*-Gal IgG antibodies in diagnosis and monitoring. This study underscores the complexity of ATL and the importance of pursuing new treatment strategies, especially considering the diversity of *Leishmania* species and individual patient responses. Further research is needed to improve the management of ATL in endemic regions.

## Materials and methods

### Ethical aspects

This study was approved (opinion number 5.086.431/2021) by the Research Ethics Committee of the Universidade Estadual de Montes Claros (CEP/Unimontes) and accredited by (Comitê Nacional de Ética em Pesquisa (CONEP).

### Study population area

This was a retrospective clinical study that used a quantitative approach. Thirty patients with a clinical and laboratory diagnosis of TL were followed up between March and December 2022, encompassing a period before and during treatment recommended by the Ministry of Health. This study was conducted in Montes Claros, located in the Upper Middle São Francisco Basin in southeastern Brazil, north of Minas Gerais. The municipality has an estimated population of 417 478 inhabitants and is located at a latitude of 16° 43′ 41″, longitude of 43° 51′ 54″ and an altitude of 638 m. The ATL-infected study group consisted of 30 patients residing in areas endemic for ATL and treated at the Reference Center for Infectious Diseases (CERDI) of Policlínica Alto São João de Montes Claros, Minas Gerais, with 1 or more persistent *Leishmania* lesions. All patients considered cases of ATL were confirmed by clinical and laboratory diagnosis (direct or molecular examination) according to the recommendations of the Ministry of Health. The inclusion criteria were patient access during the study period, patients of both sexes, aged 18 years and over, of any ethnicity, diagnosed with ATL and signed the informed consent form. Pregnant women, children and impaired individuals were excluded. Uninfected individuals: samples from 6 healthy volunteers (not infected by ATL and Chagas disease) living in an area endemic for ATL.

### Epidemiological analysis and clinical data

After the appointment, the study information was explained to the patients, and if they agreed, the signature in the TCLE was obtained, and the questionnaire was administered. Additional information was obtained from medical records and anamnesis sheets. The following epidemiological variables were analysed: sex, age, origin, education, occupation, exposure to rural areas, comorbidities, clinical form of ATL and number, location and duration of lesions. The patient was then forwarded to a biological material collection room upon medical request.

### Biological samples

Whole blood (4 mL) was collected from patients diagnosed with cutaneous Leishmaniasis at the Reference Center for Infectious Diseases (CERDI) at the Polyclínica Alto São João de Montes Claros, Minas Gerais. Among the 30 patients positive for ATL, blood was collected from 12 individuals after being diagnosed with ATL and after treatment. Eighteen patients did not complete the treatment at the end of the study period. We were also unable to follow the patients past the study period; therefore, it was impossible to verify the presence of healing of the lesions over time. Blood was collected by a qualified professional using the venipuncture technique. Sterile needles, syringes, adult phlebotomy tourniquet with lock, collection tubes (4 mL clot activator tube, FirstLaB), dressings and cotton were used. The samples were centrifuged at 4000 RPM to separate the serum. Serum (± 2 mL) was collected using a single-channel micropipette, the clot was discarded correctly, and the samples were stored at −20°C in a freezer for later use in the detection of anti-*α*-Gal IgG by ELISA, following the protocol adapted from Brito *et al*. (2016).

### ELISA

To determine the levels of anti-*α*-Gal IgG antibodies in human sera, 96-well polystyrene ELISA plates (Sarstedt^®^) were coated with a sample of Qb virus-like particles (VLPs) bearing an average of 540 covalently attached *α*-Gal molecules, which we previously used as a highly specific and effective probe of the *α*-Gal humoral immune response (Brito *et al*., 2016). These polyvalent particles, denoted VLP-Q*β*(*α*-Gal)_540_, were used at a concentration of 50 ng mL^−1^ in phosphate saline buffer pH 7.6 (PBS), incubated overnight at 4°C. The treated wells were then blocked with 1% fetal bovine serum (BSA) (Sigma-Aldrich) in PBS for 50 min at 37°C and washed. The resulting antigen-coated plates were sequentially incubated with 50 *μ*L of sera (in duplicate) at a 1:100 dilution in PBS containing 1% BSA at 37°C for 90 min, washed and incubated with secondary anti-human IgG antibody conjugated with horseradish peroxidase (HRP) (1:1000 dilution) in PBS for 30 min at 37°C. Plates were washed 5 times after each incubation step with PBS/0.05% Tween solution and then dried by inversion on an absorbent paper. The HRP reaction was developed using 100 *μ*L of peroxidase substrate SigmaFast^®^ OPD (*o*-phenylenediamine) (Sigma-Aldrich), incubated at 37°C for 30 min at room temperature, and protected from light. Addition of 4N sulphuric acid stopped the reaction. Absorbance was measured using a Multiskan GO spectrophotometer (Thermo Scientific) at a wavelength of 490 nm (MOURA *et al*., 2017).

### Statistics

Statistical analyses were performed using the GraphPad Prism software version 9.5.1 (733) (© 2023 GraphPad Software, Irvine, CA, USA). The serum ELISA results were normalized as previously described, using a 1-to-10 scale between the minimum and maximum values. (Brito *et al*., 2016) After demonstrating data normality using the Kolmogorov–Smirnov test, analysis of variance (one-way ANOVA) was performed, followed by the Bonferroni post-test to determine specific differences between groups. The normal distribution of data was verified using the Shapiro–Wilk test, and the non-parametric Kruskal–Wallis test was used for comparative analysis. Differences between groups were evaluated using the Duun–Bonferroni post-hoc method and were considered significant at *P* < 0.05.

## Results

### Study subjects

**Figure 1.**
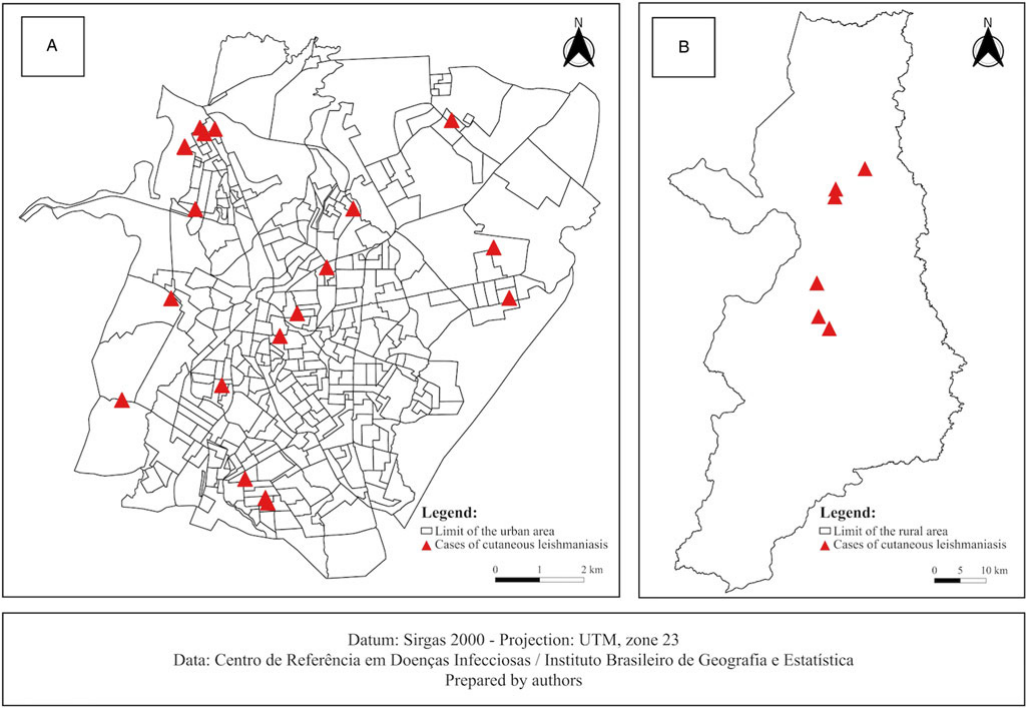
Spatial distribution of ATL cases in household units in (A) urban areas and (B) rural areas in the municipality of Montes Claros, Minas Gerais, Brazil.

As males are generally more susceptible to leishmaniasis than females (Dahal *et al*., 2021), we randomly recruited 18 males and 12 females diagnosed with ATL (*L. braziliensis* and *L. infantum*) for this study, ranging in age from 22 to 88 years (mean age, 53 years). Fourteen patients (47%) declared themselves brown (Table 1). A predominantly urban occurrence profile (70%) was observed for housing in both urban and rural areas (Fig. 1A, B). However, among these patients, 17 (57%) declared having visited the rural environment at some point before noticing the initial symptoms or wounds (Table 1).

**Table 1.** Sociodemographic profiles of patients with ATL in this study

Sociodemographic variables	Sex		Total
	Male 12 (40%)	Female 18 (60%)	30
Age			
22–57	8	10	18 (60%)
60–88	4	8	12 (40%)
Self-declared colour or race			
Asian	2	0	2 (6.66%)
Black	3	8	11 (36.66%)
White	7	10	17 (56.66%)
Native	0	0	
Residence			
Urban	8	13	21 (70%)
Rural	4	5	9 (30%)
Education			
Illiterate	2	2	4 (13.33%)
Elementary school	4	6	10 (33.33%)
High school	4	9	13 (43.33%)
College	2	1	3 (10%)
Occupation			14 (13.33%)
Domestic activities^a^	9	5	
Administrative or commercial activities	1	7	8 (26.66%)
Agricultural/rural worker	1	5	6 (20%)
Other^b^	1	1	2 (6.66%)

a Domestic activities: housewife, home secretary, cleaning lady, retirees, pensioners and unemployed.

b Others: psychologist, event promoter.

*Source:* Prepared by the author from information obtained from the Reference Center for Infectious Diseases (Also São João Polyclinic) in Montes Claros, Minas Gerais.

### Clinical manifestations of *Leishmania*

The variety of clinical symptoms associated with cutaneous leishmaniasis is similar to those of leprosy or tuberculosis; the type and strength of the host's immune response is a useful comparator. The immunological reaction to cutaneous leishmaniasis depends on a variety of host immune factors, as well as on the distinctions between infected *Leishmania* species. Knowledge about the parasite and protective measures against the disease also play a large role, as inadequate education on this subject often results in a late search for medical assistance. Among the patients, lesions existed from 1 to 24 months before the first medical appointment (Table 2), with the disseminated form having the longest duration. Among these patients, lesions appeared localized (66.6%), disseminated (30%) and mucocutaneous (3.33%) (Table 3). Thirteen patients (43.3%) had a single wound and the remaining 17 (56.7%) had multiple wounds. In general, ulcers are painless; however, 30% of patients experience pain. Eighty percent of the patients had lesions smaller than or equal to 3 cm in diameter, most frequently in the lower limbs.

**Table 2.** Time interval between symptom onset and the patient's first appointment

*Lesion time (months)*	*Sex*		*Total*
	Female 12 (40%)	Male 18 (60%)	30 (100%)
*1*	0	1	1 (3.3%)
*2*	5	4	9 (30%)
*3*	3	4	7 (23.3%)
*4*	1	2	3 (10%)
*5*	1	2	3 (10%)
*6*	1	3	4 (13.3%)
*9*	0	1	1 (3.3%)
*12*	1	0	1 (3.3%)

**Table 3.** Appearances of lesions in patients with ATL

Features	Sex		Total
	Female	Male	
Clinical form	12 (40%)	18 (60%)	30 (100%)
Localized	9	11	20 (66.6%)
Disseminated	3	6	9 (30%)
Mucocutaneous	0	1	1 (3.33%)
Lesion			
Single	4	9	13 (43.3%)
Multiple	8	9	17 (56.6%)
Size			
⩽ 3 cm	11	13	24 (80%)
⩾ 3 cm	1	5	6 (20%)
Pain			
Yes	2	7	9 (30%)
No	10	11	21 (70%)
Site			
Face	0	2	2 (6.6%)
Upper limbs	3	6	9 (30%)
Lower limbs	8	6	14 (46.6%)
Upper limbs and face	0	1	1 (3.3%)
Upper and lower limbs	1	3	4 (13.3%)

### Identification and treatment of *Leishmania* spp.

**Figure 2.**
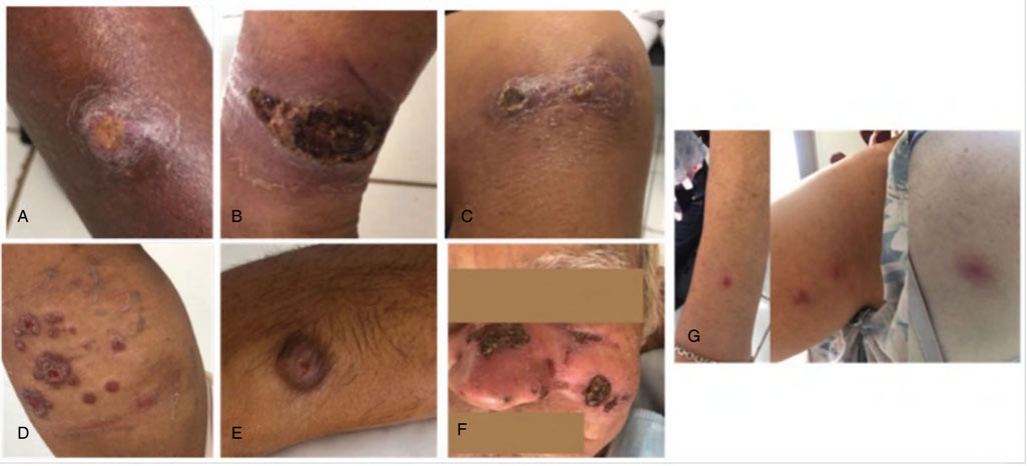
Leishmaniasis lesions. (A) *L. braziliensis*, localized form on the leg. (B) *L. braziliensis*, localized form on the posterior part of the leg. (C) *L. braziliensis*, disseminated form throughout the body. (D) *L. braziliensis*, disseminated on the arm and forearm. (E) *L. braziliensis*, localized on the arm. (F) *L. braziliensis*, mucocutaneous form on the face and nasal mucosa. (G) *L.* infantum lesions.

Seven of the 30 patients participated in a companion study to identify the infecting species of *Leishmania* using PCR/RFLP and genomic sequencing; the results are shown in Table 4. *Leishmania braziliensis* was found in all but 1 patient, of which 3 had the localized form, 2 disseminated and 1 cutaneous/mucosal form (Fig. 1A–F). *Leishmania infantum* was identified in 1 patient who had 4 ulcerated granulomatous lesions (Fig. 1G). The patient was initially treated with miltefosine with no improvement in the lesions; intralesional meglumine antimoniate was administered as a second treatment (Fig. 2).

**Table 4.** *Leishmania* spp. and patient treatment

Patient	Sex	Age	Clinical form^a^	Species^b^	Size and number of lesions	Time^c^	Treatment^d^
1	F	62	L	*inf.*	Back, arm, and forearm (4)	12	Miltefosine + MA intralesional
2	M	71	L	*braz.*	Leg (1)	3	MA intralesional
3	F	58	L	*braz.*	Leg (1)	3	Miltefosine
4	F	52	D	*braz.*	Whole body (8)	2	MA intravenous
5	M	49	D	*braz.*	Arm (10)	24	Miltefosine
6	M	77	M	*braz.*	Face and nasal mucosa (1)	9	Amphotericin B
7	M	48	L	*braz.*	Arm (1)	6	MA intralesional

### Evaluation of anti-*α*-Gal IgG antibody production in leishmaniasis patients

**Figure 3.**
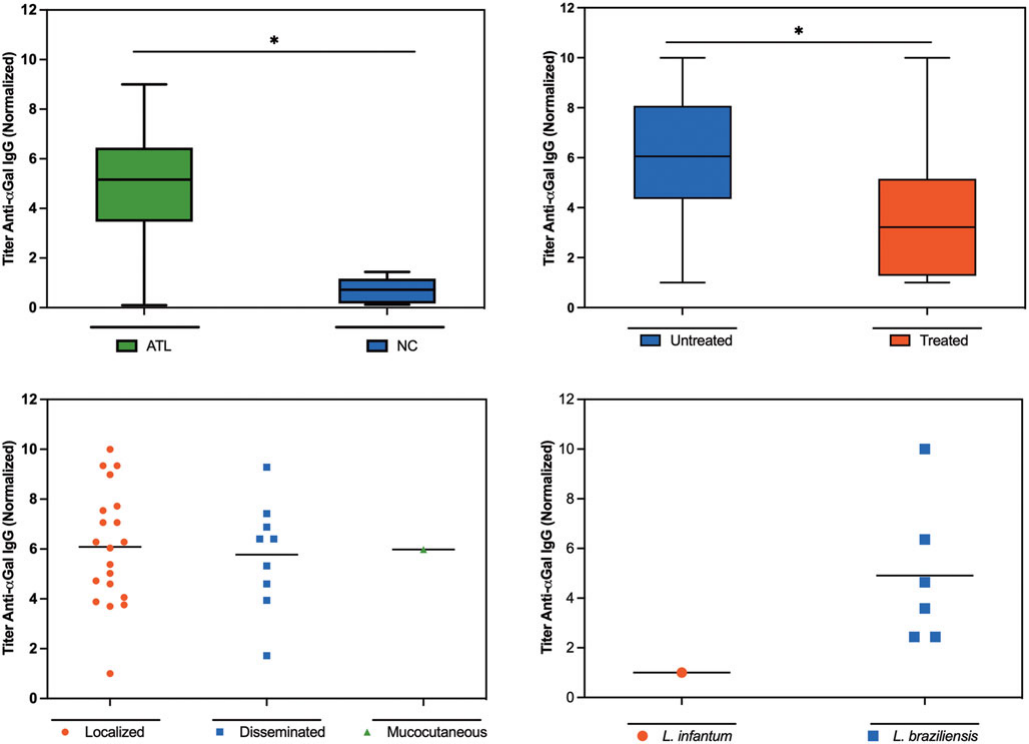
Anti-*α*-Gal IgG immune response. (A) Patients diagnosed with ATL (*n* = 30 vs healthy subjects (*n* = 6, all negative for ATL, visceral leishmaniasis and Chagas disease). (B) Anti-*α*-Gal IgG levels in the 12 patients before and after treatment. (C) Anti-*α*-Gal IgG levels in 30 infected patients separated by the clinical form. (D) Anti-*α*-Gal IgG levels in 7 patients for whom Leishmania species were identified. Sera were collected after the confirmatory diagnosis of ATL and diluted 1/100. Results were normalized. (Brito *et al*., 2016) Statistically significant differences are indicated by * (*P* < 0.05).

The level of anti-*α*-Gal IgG antibodies was found to be significantly higher in ATL-positive patients than in healthy patients (Fig. 3A). IgG levels were also measured in 12 patients after treatment, and the anti-*α*-Gal response was significantly diminished by treatment (Fig. 3B, *P* = 0.0019). We did not have sufficient data points to make meaningful distinctions between the 4 types of treatments currently recommended by the Ministry of Health. No significant differences were observed in the levels of anti-*α*-Gal IgG antibodies among the ATL-infected patients with different clinical forms (Fig. 3C). The lone patient with an identified *L. infantum* infection displayed a lower anti-*α*-Gal response (Fig. 3D) than in those infected with *L. braziliensis*, although statistical significance could not be assigned.

## Discussion

ATL is a serious public health problem and is considered by the WHO as one of the central parasitic diseases (Torres-Guerrero *et al*., 2017; de Vries and Schallig, 2022). Patients in this study were treated in the customer service specialized (CSE) care service at the Alto Policlínica São João de Montes Claros-MG outpatient clinic, which provides exclusive care to patients with tegumentary leishmaniasis. Established by the National Humanization Policy (NHP) of Brazil, this health service is the first provided to patients with leishmaniasis by health professionals, followed by clinical evaluation. Here, most ATL was observed in male patients of working age, self-declared brown, a profile observed in previous studies (Dahal *et al*., 2021; Ursine *et al*., 2023). It is believed that greater exposure, mainly due to occupational factors and hesitation in seeking health services, contributes to greater vulnerability in this cohort (Armijos *et al*., 1997; Pinto *et al*., 2020). Furthermore, due to biological (hormonal) factors, there is strong evidence of a greater predisposition of men to develop the disease (Lockard *et al*., 2019). Differences in parasitic load and clinical manifestation have been described according to gender under experimental conditions (Travi *et al*., 2002; Rodriguez *et al*., 2018).

The ATL presented a predominantly urban occurrence profile, different from the one observed in most Brazilian regions where the occurrence profile is rural (Marchi *et al*., 2019). In this study, it was noted that most residents of the urban environment frequented the rural environment. However, due to uncontrolled urbanization, deforestation and increasing human contact with wild environments, urbanization of the disease has been observed in studies previously carried out in the same municipality (Cardoso *et al*., 2019; Ursine *et al*., 2021). Due to the peculiar epidemiological characteristics of ATL, strategies for control must be flexible and distinct, suited to each region or particular focus and considering environmental, animal and human health, as well as the interaction between professionals from different areas of knowledge for health promotion (Semenza and Zeller, 2014). As in other studies, patients affected by ATL in this study were delayed in seeking health services and, consequently, starting treatment.

The time of evolution of ATL symptoms determines the severity of the disease. Delays in diagnosis and treatment initiation may lead to complications and irreversible sequelae in patients. We observed patients with severe, persistent lesions who did not respond well to treatment, which may be associated with several factors, including the time since disease onset. Even though infection of the mucosa is less frequent than that of the skin, involvement of the oral and nasal mucosa is usually more severe. Furthermore, the advanced and mutilating forms of the disease can lead to psychological, social and behavioural harm (Diniz *et al*., 2011). Regarding the clinical form, the localized cutaneous form was predominant, as observed in other studies, and this form is responsible for more than 90% of cases in Brazil (Gosch *et al*., 2017; Grangeiro Junior *et al*., 2018). The areas most affected by sandfly bites are the most exposed areas of the body, predominantly the lower limbs, followed by the upper limbs. Localized lesions in the upper limbs, head, and trunk tend to evolve and heal more quickly than those located in the lower limbs (Pinart *et al*., 2020). However, this profile was not a general rule in the evaluated patients, as 1 patient from the study remained with an arm injury for 24 months, and another with arm injuries and a forearm did not respond well to the first therapeutic regimen, requiring a second treatment.

Immunosuppression caused by human immunodeficiency virus (HIV), associated with infections caused by the protozoan *Leishmania*, can lead to disease progression. It is recommended to perform serology for HIV in all patients with ATL (Lindoso *et al*., 2016). In this study, all patients were tested for HIV; none had *Leishmania*/HIV co-infection. As it is also an endemic area for Chagas disease, we verified that no patient was affected by *Trypanosoma cruzi*. However, some comorbidities were observed, and systemic arterial hypertension (SAH) was prevalent among the studied patients (40%). SAH is one of Brazil's most important non-transmissible chronic diseases (NTCD) and influences the choice of therapy for ATL.

Intralesional application of meglumine antimoniate (Glucantime) is the most prevalent treatment for ATL and was shown to be effective in 87% of cases in a 2018 study by Ramalho *et al*. (2018). This route of administration was adopted by the Ministry of Health in 2017 through the tegumentary leishmaniasis manual, noting that the treatment modality should be dictated by the clinical form, with the support of laboratory diagnosis and complying with the criteria established for each situation. Our results also highlighted the importance of identifying the species involved since we observed therapeutic failure in a patient infected with *Leishmania infantum*. Similar findings have been described for the treatment of the Old World species *L. major* and *L. tropica* (Chakravarty and Sundar, 2010).

In 2014, a group of researchers reported that patients infected with *Leishmania* spp. in the Old World (*L. major* and *L. tropica*, different species than those found in Brazil) had significantly higher levels (up to 9-fold) of anti-*α*-Gal IgG compared to healthy control subjects (Al-Salem *et al*., 2014). The same study presented the intriguing observation that cured individuals showed higher levels of anti-*α*-Gal IgG antibodies than healthy individuals, suggesting that after chemotherapy, more *α*-Gal epitopes in intracellular amastigotes may be exposed to the host's immune system, leading to a greater B-cell response and a considerable increase in anti-*α*-Gal titre (Ramirez and Guevara, 1997; Al-Salem *et al*., 2014). We note, however, that low parasitaemia can occur in scars of patients cured of ATL. The results described here show a similar increase in anti-*α*-Gal levels in infected *vs* healthy patients but a decrease in anti-*α*-Gal IgG antibodies after treatment. The difference with the Old-World study may be due to several factors, including the fact that Brazilian patients were not completely cured and that the treatment mechanism did not involve a direct immunological component. In these cases, we suggest that treatment induces a drop in parasitaemia and, therefore, in the *α*-Gal epitope stimulus. Indeed, we observed considerable decreases in anti-*α*-Gal antibody levels in patients treated for Chagas disease in both published (Andrade *et al*., 2004; Brito *et al*., 2016) and recent unpublished studies by our group.

Moura *et al*. (2017) demonstrated that vaccination with *α*-Gal in a model of *α*-galactosyltransferase knockout mice (which mimic the anti-*α*-Gal immune response observed in humans) protected them against *L. amazonensis* and *L. infantum*, preventing parasitic infection in liver and spleen (Moura *et al*., 2017). These results support the possible efficacy of *α*-Gal immune response against leishmaniasis in humans. It was also noted that the expression of the *α*-Gal epitope is significantly lower in *L. infantum* than in the thermotropic species *L. amazonensis*, which is consistent with the finding of lower anti-*α*-Gal titre in the patient in this study infected with *Leishmania infantum*. Indeed, there is a lack of reports of the presence of the *α*-Gal epitope in other species of *Leishmania* spp. present in Brazil: our findings here of elevated anti-*α*-Gal IgG antibodies in 30 patients in an urban Brazilian setting strongly suggest that this is true concerning *L. braziliensis*.

We also noted that a correlation exists between the ABO blood type and susceptibility to certain infectious diseases, including malaria (Rispens *et al*., 2013; Cabezas-Cruz *et al*., 2017); blood group A and B antigens resemble *α*-Gal. As most of the patients in our study were in group O, we had no opportunity to incorporate this factor into our analysis.

It has been reported that *α*-Gal antigens on the pathogen surface can play an important role during infection (Yilmaz *et al*., 2014; Cabezas-Cruz and de la Fuente, 2017; Cabezas-Cruz *et al*., 2017; Pacheco *et al*., 2020), and a complementary relationship appears to emerge for the anti-*α*-Gal immune response. Yilmaz *et al*. (2014) showed that the production of anti-*α*-Gal antibodies is associated with protection against malaria transmission. They suggested that immunization with adjuvants that favour the production of anti-*α*-Gal IgM antibodies might protect against malaria (Yilmaz *et al*., 2014). In an investigation of anti-*α*-Gal IgG antibody levels in COVID-19, the development of interventions with probiotics based on commensal bacteria with *α*-Gal epitopes was proposed to modify the microbiota and increase *α*-Gal-induced protective immune response (Urra *et al*., 2021). Finally, patients infected with *T. cruzi* showed high levels of anti-*α*-Gal in the acute and chronic phases of Chagas disease (Avila *et al*., 1988; Travassos *et al*., 1988; Gazzinelli *et al*., 1991). Anti-*α*-Gal antibodies are known to bind *α*-Gal present on the *T. cruzi* surface and induce complement-mediated lysis of the parasite, suggesting that anti-*α*-Gal contributes to host protection (Towbin *et al*., 1987; Gazzinelli *et al*., 1991).

In this study, we conclude that by monitoring 30 ATL-infected patients, our investigation reaffirmed the significance of serum IgG antibody levels against the *α*-Gal trisaccharide motif as a potential marker of *Leishmania* susceptibility. The significantly elevated anti-*α*-Gal antibody levels observed in ATL-infected individuals compared with healthy subjects highlight the promising utility of this biomarker in diagnostic and monitoring applications. Furthermore, the predominant use of meglumine antimoniate in treatment, when administered to most patients, yielded limited symptom improvement, suggesting potential challenges in current therapeutic approaches. Nevertheless, post-treatment analysis revealed a significant decrease in IgG anti-*α*-Gal antibodies in a subset of patients, indicating a potential correlation between the treatment response and antibody levels.

Notably, the identification of *Leishmania* species revealed a predominance of *Leishmania braziliensis* infections exhibiting heightened anti-*α*-Gal responses in contrast to a single case of *Leishmania infantum* infection. This underscores the importance of considering *Leishmania* species diversity in developing tailored treatment strategies and emphasizes the urgent need for future research focusing on human vaccines and innovative therapeutic interventions.

## Data Availability

N/A.
